# Factors associated with children’s HIV- positive status disclosure in Wolaita Zone, Southern Ethiopia: a cross-sectional study

**DOI:** 10.1186/s13052-022-01287-6

**Published:** 2022-06-06

**Authors:** Belete Gelaw Walle, Chalie Marew Tiruneh, Tigabu Dessie, Nigusie Selomon, Amare Kassaw, Bogale Chekole, Moges Wubneh, Tadele Lankrew, Wubet Alebachew Bayih

**Affiliations:** 1 Department of Pediatrics and Child Health Nursing, School of Nursing, College of Health Science and Medicine, Wolaita Sodo University, P.O.Box 138, Wolaita Sodo, Ethiopia; 2 Department of Pediatrics and Child Health Nursing, College of Health Sciences, Debre Tabor University, Debre Tabor, Ethiopia; 3 Department of Nursing, College of Medicine & Health Sciences, Wolkite University, Wolkite, Ethiopia; 4 Department of Adult Health Nursing, College of Health Sciences, Debre Tabor University, Debre Tabor, Ethiopia; 5 Department of Adult Health Nursing, Wolaita Sodo University, Wolaita Sodo, Ethiopia; 6 Department of Maternal and Neonatal Nursing, College of Health Sciences, Debre Tabor University, Debre Tabor, Ethiopia

**Keywords:** ART, Children, Disclosure, HIV/AIDS, Ethiopia

## Abstract

**Background:**

Children’s HIV-positive status disclosure is an essential component of chronic care & long-term disease management. The modalities of status disclosure are complex and vary across different communities. Although data from various settings are necessary to overcome this problem, evidence is limited, specifically in the in the study areas. Therefore, this study aimed to assess the prevalence of HIV-positive status disclosure and associated factors among children on antiretroviral therapy (ART).

**Methods:**

A mixed-method, facility-based study was conducted: among 203 caregivers with children in Wolaita Zone, Southern Ethiopia. We used in-depth interviews for qualitative data in addition to structured questionnaires. Simple random sampling for quantitative and purposive sampling for qualitative parts was applied. We used Content or Thematic analysis for qualitative and Binary logistic regression for quantitative.

**Results:**

All the caregivers were interviewed and majorities (84.7%) of them were female. One hundred twelve children (55.2%) were below 12 years of age. In this study, the overall prevalence of children with HIV-positive status disclosure was 46.8%. Being 12 or more years of age (AOR = 7.5, 95% CI: 2.9–15.6), duration on ART 72 or more months (AOR = 3.8, 95% CI: 1.7–6.7), death of the parent (AOR = 2.0, 95% CI: 1.1- 3.8), and having follow up in the hospital (AOR = 2.1, 95% CI: 1.3–4.7) were associated with disclosure. Being an immature child was the commonest reason cited by caregivers for non-disclosure. Frequent questions by the child about why they are taking drugs, for better self-care, and treatment adherence were the commonest reason of caregivers for disclosing their children’s HIV-positive status.

**Conclusion:**

In our study, children’s HIV-positive status disclosure was significantly low. This study identified different factors (Health facility, children, and caregivers-related factors) as the main reason for disclosing children’s HIV-positive status. Hence, health care workers should give special attention to children’s HIV-positive status disclosure, which helps to increase the effectiveness of treatment and prevent further HIV transmission.

**Supplementary Information:**

The online version contains supplementary material available at 10.1186/s13052-022-01287-6.

## Background

Globally, Pediatric human immunodeficiency virus (HIV) infection continues to be a public health concern and challenge [1]. At the end of 2019, approximately 38.0 million people were living with HIV worldwide, of which 1.8 million were children (age 0–14 years) [2]. The Sub-Saharan African (SSA) countries were suffering from the burden of HIV- infection. By the end of 2018, in Ethiopia, there were an estimated 56,514 HIV-infected children (2,994 newly infected) [1].

American Academy of Pediatrics and the World Health Organization (WHO) strongly recommends the gradual process of giving age-appropriate information to HIV-infected school-age children by considering the child’s cognitive and emotional development [3, 4]. The African Network for the Care of Children Affected by HIV/AIDS (ANECCA) recommends that pediatric HIV disclosure start as early as age between 5 to 7 years old [5]. HIV-positive status disclosure remains a hindrance in the fight against HIV spreading in sub-Saharan Africa [3, 6].

Disclosure helps the child gain knowledge of their HIV-positive status [7, 8]. Studies showed that they are more likely to adhere to antiretroviral therapy (ART) [9] if children live with HIV know their HIV status[9]. There are several findings of the clinical and psychosocial benefits of the HIV-positive status disclosure to infected children, which helps to improve the quality of life of people infected and affected by the HIV disease[7, 9–11]. The World Health Organization (WHO) reported that the knowledge about their disease enables individuals to make safe & healthy life choices about relationships, sex, and reproduction [12].

In Ethiopia, research was conducted on children’s HIV-positive status disclosure-based up on previous guidelines [13–15]. Factors like a history of ART interruption and restart, caregiver's HIV status, caregiver’s duration on ART, place of ART follow up, and disclosure status of caregivers, which contribute to the occurrence of low level of disclosure, was infrequently included in the former studies. Most of the prior studies were done at the referral h[13, 14]. This study encompasses health centers that cover the majority of health facilities and provide pediatric ART services in Ethiopia. As to our knowledge, there were no studies about HIV-positive status disclosure and its associated factors among children living with HIV in the study area. Current and up-to-date evidence regarding children’s HIV-positive status disclosure is essential for policymakers and clinicians to take appropriate measures. Therefore, by aiming to fill these gaps and increase the body of knowledge about HIV-positive status disclosure, this study was conducted to determine the prevalence and the contributing factors of HIV- Positive status disclosure among children attending ART clinics in Wolaita Zone public health facilities.

## Methods

### Study settings, design, and period

A facility-based, mixed-method study was conducted from July to September 2021 among HIV-infected children on ART. The study was carried out in Wolaita Zone public health facilities which provide pediatric ART services. Wolaita Sodo is the capital city of Wolaita Zone, located in Southern Ethiopia, which is 338 km away from Addis Ababa. We did this research in six public health facilities (Bodity Health Center, Areka Health Center, Sodo Health Center, Humbo Primary Hospital, Bitena Primary Hospital, and Wolaita Sodo University Comprehensive Specialized Hospital).

### Study participants, sample size, and sampling technique

All the caregivers of HIV-positive children (6 to 18 years) who have been taking ART in the pediatric ART clinics were eligible. However, children who came alone or had no caregivers or parents to undertake the consent, caregivers diagnosed to have a mental health problem or with other serious illnesses were not eligible.

The minimum required sample size was determined using a single and double population proportion formula. While computing the sample size for the first objective, the following statistical assumptions were considered 95% confidence level (CI), Proportion = 43.6% taken from a study conducted in western Ethiopia [16], the margin of error = 5%, and the value of Zα/2 = 1.96, which is the corresponding Z score of 95% confidence interval (CI).

$$\mathbf n=\frac{({\mathbf Z\mathbf a\boldsymbol/\mathbf2)}^{\mathbf2}\mathbf p(\mathbf1-\mathbf p)}{\left(\mathbf d\right)^{\mathbf2}}=\frac{({\mathbf1\boldsymbol.\mathbf{96})}^{\mathbf2}\ast\mathbf0\boldsymbol.\mathbf{436}(\mathbf1-\mathbf0\boldsymbol.\mathbf{436})}{\left(\mathbf0\boldsymbol.\mathbf{05}\right)^{\mathbf2}}=\mathbf{378}$$

where, *n* = the required sample size, Zα/2 = Standard normal variation for type 1 error, *p* = prevalence (0.436) & d = Margin of sampling error tolerated (0.05).

So, the final estimated sample size was 203 by considering a 10% non-response rate and using the population correction formula as the total study population was 339 (below 10,000) in the study area.

This study was carried out in six randomly selected public health facilities. We found the list of participants who had regular ART follow-up visits from the electronic database and their registration book in each health facility. Sampling-frame was prepared based on the child’s medical registration number (MRN) from documented files of each health facility. After determining the total number of children in each health facility, We proportionally allocated to each health facility. Lastly, we used a simple random sampling method to select the proportionally allocated study participants. We used a purposive sampling technique to select participants for the qualitative study.

### Data collection tool and procedure

We developed quantitative and qualitative data collection tools from different literature. The questionnaire was prepared in English and translated to the local language translated back to English to ensure consistency. Quantitative data were collected using structured questionnaires through face-to-face interview**s** and supplemented by a chart review. Some HIV related data were extracted from the child’s medical record in the ART units of each health facilities. Twelve trained nurses have participated in the quantitative data collection. We conducted a pretest out of the study area before the actual data collection. We deliver the training to data collectors and supervisors on the objectives, methods of data collection, and how to obtain informed consent. Additionally, the data collectors gave a brief introduction to study participants during the data collection process. We conducted continuous supervision during data collection time.

Qualitative data collection was conducted concurrently with quantitative data collection. We translated and transcribed the in-depth interviews (IDI) (from the caregivers and health care workers). The principal investigator carried out an audio-record interview. The audio record aimed to triangulate the issues raised by the caregivers regarding disclosing or not disclosing and suggestions for improving disclosure (See Supplementary file 1).

### Operational definitions

Disclosure: when the child knows their HIV/AIDS diagnosis status regardless of who told the child [17].

A caregiver: is a person who is knowledgeable about the child’s HIV care, responsible for the well-being of the children, and who brought the child to the clinic [18].

Adherence: attends to their regular clinical follow-up care, periodic laboratory monitoring, and avoids practices that interfere with treatment effectiveness [18].

### Data management and statistical analysis

Data were entered into Epi Data Version 3.1 software packages and exported to IBM Corp. Released 2017. IBM SPSS Statistics for Windows, Version 25.0. Armonk, NY: IBM Corp for further analysis. We present the descriptive data via tables and figures.

Hosmer–Lemeshow goodness test was applied to check the model fitness, and the logistic regression method was applied to select significant variables. Bivariate analysis was done to identify the candidate variables for multivariable analysis. Then, Variables with *p*-values < 0.25 were eligible for the multivariable analysis. We used Variance Inflation Factor (VIF) to check the multicollinearity. Variables with *p*-values < 0.05 in the multivariable analysis were considered statistically significant factors. Finally, an odds ratio with 95% CIs was used to assess the strength and the direction of association between outcome and study variables.

We transcribed and translated the recorded data from in-depth interviews into English word by word. We used thematic analysis for the qualitative data. We used the principles of content analysis, applying appropriate codes, sorting data, and looking for differences and similarities to analyze the transcription. Lastly, we put representative quotes on the range of summarized data.

## Results

### Socio-demographic characteristics of caregivers

A total of 203 study participants were eligible for this study. A majority (84.7%) of caregivers were female, and (70.9%) of caregivers were HIV positive. Among the caregivers, 38.4% had no formal education, and nearly half (48.8%) of the caregivers were married. The mean age of caregivers was 34.93 years (SD =  ± 4.87). Most (86.7%) of caregivers were first-degree relatives (See Supplementary file 2: Table S1.).

### Socio-demographic characteristics of the children

Nearly half of the children (54.2%) were females. Almost all (89%) of the children had started formal education, and about 87.7% of the children live with their parents. In addition, about 34.5% had lost any of their parents, and the majority (89.2%) of children had good ART adherence levels. The mean age of children was 11.5 years (SD: 2.69). The mean age of children at HIV their diagnosis was 3.92 years (SD ± 2.5). The majority (82.8%) of participants had no history of ART interruption (See Supplementary file 3: Table S2).

### Prevalence of HIV positive status disclosure

A total of 95 HIV-infected children knew their HIV-positive status. The overall prevalence of disclosure among HIV-infected children was 46.8% (95% CI: 39.7, 54.6%).

### Factors associated with HIV-positive status disclosure

Bivariable and multivariable logistic regression analyses were applied. In the multivariable logistic regression analysis, types of health facility, child age, death of any of their parent, and child’s duration on ART were factors significantly associated with HIV positive status disclosure. Thus, children with age 12 and above years were more likely (adjusted odds ratio [AOR] = 7.54, 95% CI: 2.87–15.62) to know their HIV-positive status. Similarly, those children who took ART for 72 or more months were more likely (AOR = 3.84, 95% CI: 1.65–6.72) to know their HIV-positive status with reference to their counterparts. Moreover, when compared to children who had both parents alive, children who had lost one of their parents were more likely (AOR = 1.96, 95% CI: 1.05–3.84) to be disclosed their HIV-positive status. Regarding types of health facility, children’s HIV-positive status disclosure was nearly 2 times (AOR = 2.13, 95% CI: 1.33—4.67) higher to occur in those children who have ART follow up at the hospital level compared to their counterparts (See Supplementary file 4: Table S3).

### Findings from the qualitative study

The majority of the caregivers said that children’s HIV-positive status disclosure is necessary for treatment effectiveness. Hence, after HIV positive status disclosure, children have good ART adherence levels, improve their awareness to live with HIV, and decrease worries and confusion. All the study participants agreed upon the importance of a child’s HIV positive status disclosure. HIV-positive status disclosure should be applied when the child becomes mature, and avoid disclosing for the young children since they cannot understand whenever we are talking about HIV/AIDS. We had asked both caregivers and health care workers regarding their experience with a child’s HIV-positive status disclosure. One of the caregivers (mother) talked about her disclosure experience as follows “To disclose HIV positive status of our child, first I had discussed with my husband for a week. Then I had an open discussion with my child about the mode of transmission, treatment, and prevention of HIV/AIDS. I am HIV- infected and taking the drug which helps me to carry out my activity of daily living. You are also infected with this disease which is acquired during labor when I gave birth. You are taking a drug to keep you strong and healthy. If you take your drug properly, you can do everything effectively without any limitations what other healthy individuals can do.” The other caregiver put her experience as follows “I had lost my strength to disclose my child’s HIV positive status. I fear my child’s worry following disclosure and I feel that I do not have enough information about HIV/AIDS to convince the child. Finally, I decided to go to the health facility with my husband to get assistance from health care workers. Then, we (mother, father, and health care workers) had given information about the disease. But, the child feels confused and crying. After disclosure, the child refused us to take drugs for a few days. After a few days, she starts to take care of herself and take drugs properly.”

The most common feelings experienced by children during disclosure of their HIV-positive status are crying to their caregivers, refusing to go to school, and feeling anxiety for a week. Despite it being rare, children had gotten difficulty to have slept for a few weeks and attempted to harm themselves following their HIV-positive status disclosure. Conversely, they withdraw from such unusual feelings with the help of their caregivers and health care workers. After fourteen years, some children experience unsafe sex, and others try to practice safe sexual practices (ART nurses).

Disclosing the child’s HIV-positive status is also carried out through other HIV-positive children who know their status. But, the implementation of the peer announcing strategy is rare. A child who knows their HIV-positive status says I am living with HIV and attending may school effectively. At this time, those previously disclosed children will share their experiences with the newly informed child. The child will take others as a model and seeks to achieve their vision by taking drugs properly (ART nurses).

### Reasons for HIV- positive status disclosing or not disclosing

The majority of the caregivers have stated the following reasons to explain why they are taking medications (for treatment of pneumonia, common cold, parasite, and germs). Frequent questions by the child about why they are taking medications were the common reason for the caregivers to decide to disclose their children’s HIV-positive status. Additionally, if the child knows their HIV-positive status, they will take medications properly and take appropriate care of themselves. This will make life easy for both the caregivers and the child. Some caregivers decided to disclose the child's HIV-positive status to prevent accidental disclosure.

The caregiver’s response to the question (why they did not disclose the child’s HIV-positive status): the child’s thought is immature, cannot understand talking about HIV/AIDS. In addition, fear of social stigma and discrimination was also another reason. The children are too young, and disclosing their HIV-positive status will make them worry and distressed. Moreover, lack of separate room, lack of specific short term training, time, and challenges to assure caregivers were challenges mentioned by health care workers to conduct disclosure.

## Discussion

In this study, the prevalence of disclosure among HIV-infected children (aged 6 to 18 years old) was 46.8% (95% CI: 39.7, 54.6%). It is in line with the finding of studies done in Southwest Ethiopia (45.6%) [19], Eastern Ethiopia (49%) [14], Western Ethiopia (43.6%) [16], and Northern parts of Ethiopia (44%) [20]. On the contrary, this finding is lower than studies conducted in Uganda (65%) and Rwanda (64%) [21, 22]. However, the finding of this study is higher than the studies done in Zambia (29.8%)[10], Ghana (23.3%)[11], Nigeria (30.9%)[23], and Namibia (33%)[7]. The possible source of variation could be due to the differences child’s age, study time, sample size, psychosocial factors (social stigma and discrimination), and caregivers’ awareness towards the importance of disclosure.

We found that different factors were significantly associated with children HIV positive status disclosure. In this regard, children aged 12 years and above were more likely to be disclosed their HIV-positive status. This finding is in line with previous studies done in Nigeria [23], Uganda [21], Tanzania [24], Ghana [25], and Ethiopia [26]. This is because children at the age of 12 years and above are mature enough to understand HIV /AIDS as they can get some information regarding this at their school. Additionally, most caregivers agreed upon the importance of HIV-positive status disclosure when the child is mature.

This study showed that children who had taken ART for ≥ 72 months were more prone to be disclosed their HIV-positive status independently for their age. This result is concordant with other reports from Uganda [27] and Ethiopia [26]. It is known that children and caregivers having long periods of ART follow-up visits can get ongoing information regarding HIV/AIDS which facilitates children’s HIV positive status disclosure. Lost any of their parents was also significantly associated with children HIV positive status disclosure. This can be explained, because those children who lost any of their parents ask their family members why their parents passed away. The majority of the parent could be dead due to HIV/AIDS-related factors. Due to this, the children may know their status.

Lastly, children who have ART follow-up at the hospital level were more likely to be disclosed their HIV-positive status. This finding is agreed with studies reported from Southwest and Northwest Ethiopia [19, 20]. This might be because, even though both hospitals and health centers are using the same guidelines, there is a difference in the diversity of health care workers, which results in a variation in detecting, counseling, and disclosure.

The results from qualitative data of this study also revealed that there were different justifications related to children’s HIV-positive status disclosure. Most caregivers approved that children’s HIV-positive status disclosure is essential for treatment effectiveness. After HIV -positive status disclosure, children’s worries and confusion will decrease. They will also have a good ART adherence level and improve their awareness to live with HIV. Conversely, the child’s age (too young) was the most common intention of caregivers not to announce HIV-positive status. This finding is supported by studies conducted in Tanzania [24], and Ethiopia [14, 16, 20].

Moreover, fear of social stigma, fear of emotional and psychological disturbance, fear of child to keep secret, and ages (too young) of the child were hindering factors mentioned by caregivers to disclose children HIV positive status. This is in line with previous studies done in Zambia [28], and Ethiopia [16, 26]. Likewise, the child’s frequent questions about why they are taking the drug every day were also the caregiver’s reason to disclose. This finding is in agreement with studies reported in Zambia [28], Nigeria [29], and Ethiopia [16, 26]. Lastly, lack of separate room, lack of specific short-term training for HIV status disclosure, time, and challenges to assure caregivers were challenges mentioned by health care workers to conduct disclosure.

### Limitations

Before interpreting the results, this study has its limitation that must be considered. Since the study was based on respondents’ answers to questions related to events happening in the previous time, there might be potential recall bias. Similarly, there might be social desirability bias as the study was based on caregivers’ information. We used secondary information to cross-check some data and minimize the above problems. Information about children’s HIV-positive status disclosure was obtained from caregivers and health care givers reports which could be biased. Since this is a cross-sectional study design, it could not establish the cause-and-effect relationship.

## Conclusion

The results of this study established that there was a low level of children’s HIV-positive status disclosure. In the study area, there were no published articles; this study will be used as baseline evidence regarding children’s HIV-positive status disclosure. Having ART follow up in the hospital, child age, death of any of their parents, and child’s duration on ART were significantly associated with children’s HIV-positive status disclosure. Caregivers challenged to disclose their children because they think that their child was too immature to understand HIV/AIDS, fear of stigma, and discrimination from the community. Additionally, children’s HIV-positive status disclosure was delayed due to health care workers believing that they lack short-term training, time, separated rooms, and challenges to reassure caregivers. So, in both the hospitals and health centers, the ART case managers and multidisciplinary teams should focus on implementing HIV-positive status disclosure counseling services for caregivers and children to improve the children’s treatment outcomes. Furthermore, working on caregivers’ knowledge and attitude and updating health care workers' skills are very important to manage challenges and increase the rate of disclosure.

## Supplementary Information

**Additional file 1.** Questionnaire 1. 

**Additional file 2:** **Table S1.** Socio-demographic and clinical characteristics of caregivers in Wolaita Zone, Southern Ethiopia, 2021 (*n*=203).

**Additional file 3**: **Table S2.** Socio-demographic and clinical characteristics of children in Wolaita Zone, Southern Ethiopia, 2021 (*n*=203).

**Additional file 4:** **Table S3.** Bivariable and multivariable logistic regression analysis of factors associated with HIV positive status disclosure among HIV–infected children in Wolaita Zone, Sothern Ethiopia, 2021 (*n*=203).

## Data Availability

All the data supporting the study findings are within the manuscript. The additional detailed raw data sets used during this study are available from the corresponding author upon reasonable request.

## Abbreviations

AHC: Areka Health Center
AIDS: Acquired Immune Deficiency Syndrome
AOR: Adjusted odds ratio
ART: Antiretroviral Therapy
BPH: Bitena Primary Hospital
BHC: Boditi Health Center
CI: Confidence Interval
COR: Crud odds ratio
HIV: Human immunodeficiency Virus
HPH: Humbo Primary Hospital
IDI: In-depth interview
SHC: Sodo Health Center
WSUCSH: Wolaita Sodo University Comprehensive Specialized Hospital
UNAIDS: Joint United Nations Programme on HIV/ AIDS
WHO: World Health Organization
