# Supplementary material for: Factors associated with children’s HIV- positive status disclosure in Wolaita Zone, Southern Ethiopia: a cross-sectional study
Source: Ital J Pediatr. 2022 Jun 6;48:86. doi: 10.1186/s13052-022-01287-6 (PMC9169329; doi:10.1186/s13052-022-01287-6)
Supplement: Supplementary file 1 — Additional file 1. Questionnaire 1. [file 13052_2022_1287_MOESM1_ESM.docx]

**Annex 3: Data collection tools**

- 1. **Quantitative Questionnaire**

**3.1.1. English Questionnaire**

Structured questionnaires prepared to collect socio demographic characteristics of the care giver and a child, clinical factors and personal factors of the care giver to assess prevalence and associated factors for HIV positive status disclosure in children.

| Part 1: Information on socio demographic characteristics of the caregiver | | | | |
| --- | --- | --- | --- | --- |
| S. No | Question | Response | Code | Skip |
| 101 | Age | …………. yr. |  |  |
| 102 | Sex | 1. Male 2. Female |  |  |
| 103 | Religion | 1.Orthodox Christian  2. Muslim 3. Protestant  4.Catholic 5.other |  |  |
| 104 | Marital status | 1. Married 2. Single  3. Divorced 4. widowed |  |  |
| 105 | Residence | 1. Urban 2. Rural |  |  |
| 106 | Level of education | 1. Didn’t read and write  2. Primary 3. Secondary  4. Diploma 5. Degree  6. Masters 7. Other |  |  |
| 107 | Relationship with the child | 1. Father 2. Mother  3.Brother 4. Sister 5. Other |  |  |

| Part 2: - Information on socio demographic characteristics of the child | | | | | | |
| --- | --- | --- | --- | --- | --- | --- |
| 201 | Age | ………. Yr. | |  | |  |
| 202 | Sex | 1.Male 2.Female | |  | |  |
| 203 | Religion | 1.Orthodox 2.Muslim  3.Protestant 4.Catholic  5.Other | |  | |  |
| 204 | The place of follow up | 1.Health center  2.Hospital | |  | |  |
| 205 | Educational Status | 1. Illiterate 2. literate  3. KG 4. Primary School (1-8)  5. Secondary School (9-12) 6. Other | |  | |  |
| 206 | Has the child lost any of his/her nucleus family members with HIV? | 1.yes 2.No | |  | | If the answer is No skip to Q No 301 |
| 207 | If the answer is yes for question No 206 whom? | 1.mother only 2.Father only  3.Brother only 4.Sister only  5.Both mother & father 6.Other | |  | |  |
|  | | | | | | |
| Part 3: Information on clinical characteristics of the care giver | | | | | | |
| S. N | Question | Response | Code | | Skip | |
| 301 | Have you tested for HIV? | 1. Yes 2. No |  | |  | |
| 302 | If the answer is yes what was the result? | 1. positve 2. Negative  3. Unknown |  | | If the answer is 2 or 3 skips to Q No 401 | |
| 303 | If the answer is 1 for whom did you disclose your positive status? | 1. To my partner 2. To my Mother  3. To my father 4. To my child  5. To my friend 6. To my brother  7. To my sister 8. To my relatives  9. To my Religious father  10. No one knows 11. other |  | |  | |
| 304 | Did you start ART? | 1. Yes 2. No |  | |  | |
| 305 | If the answer is 1 how much time is it up to now | ---------------year |  | |  | |

| Part 4: Information on clinical characteristics of the child | | | | | | | |
| --- | --- | --- | --- | --- | --- | --- | --- |
| 401 | Age at diagnosis of HIV | | ………. yrs. | |  | |  |
| 402 | Duration on ART of the child | | ………months. | |  | |  |
| 403 | History of Hospitalization | | 1.yes  2.No | |  | |  |
| 404 | With whom currently living? | | 1. Biological parents 2. Other relatives | |  | |  |
| 405 | WHO clinical staging  Of the child | | 1. stage 1 2. Stage 2  3. Stage 3 4. Stage 4 | |  | |  |
| 406 | Has a child ever affected by OIs? | | 1. yes 2. No | |  | |  |
| 407 | With Whom the child is currently living | | 1.biological parent 2. Brother  3.Sister 4.Relatives  5.other | |  | |  |
| 408 | History of ART interruption and restart | | 1. Yes 2. No | |  | |  |
| 409 | Drug Adherence | | 1.Good 2.Fair 3.Poor | |  | |  |
| Part 5: Information on HIV disclosure status among HIV positive children | | | | | | | |
| S. No | Question | Response | | code | | Skip | |
| 501 | Did the child know his/her HIV status? | Yes  No | |  | | If the answer is No skip to Q No 505 | |
| 502 | If the answer is yes for question No 501 at what age did you disclose? | At…………. yr. | |  | |  | |
| 503 | Who disclosed about the HIV status to the child? | 1.father 2.mother  3. sister 4.brother  5.relative  6.Health professional 7.Others | |  | |  | |
| 504 | Why did you decided to disclose to the child about her /his HIV status? | 1.Because the child thought to be matured  2.Repeated question by the child  3.To prepare the child to take his/her medicine  4. To take care of his/her selves and to prevent transmission unknowingly.  5. Fear of accidental disclosure. 6.others(specify) | |  | |  | |
| 505 | If you didn’t disclose why you didn’t decide to disclose? | 1.Because the child is too young  2.Fear that the child had refused to  take his/her medicines  3.Fear of stigma and discrimination  4.Fear of the child’s in ability to keep secret  5. The presence of a person in the family who is un aware of the child’s status.  6.Future relation with family will be affected  7. Fear of negative emotional and psychological consequence  8.others (specify) | |  | |  | |
| 506 | If you didn’t disclose what you told the child the reason for visiting health facility? | 1. For TB, 2. For appointment  3. For medication 4. other | |  | |  | |
| 507 | Do you think you have a responsibility for the future to disclose? | 1.yes 2. No | |  | |  | |
| 508 | Do you plan to disclose in the future about his /her HIV status to your child? | 1.yes 2.No | |  | |  | |
| 509 | If the answer in question No 508 Yes At what age the child should know his/her HIV status. | …………. yrs. | |  | |  | |
| 510 | Who should have a responsibility to disclose HIV status to the child | 1.father 2.mother  3.grand parents  4.health care provider  5.other(specify) | |  | |  | |
| 511 | Care giver Level of confidence at disclosure | 1.Confident  2.Somehow confident  3.Not all confident | |  | |  | |
| 512 | Support from the Health care provider. | 1.Yes 2.No | |  | |  | |
| 513 | Type of disclosure | 1.Compelete 2.Partial  3.Deception | |  | |  | |
| 514 | How you disclose to the child? | 1.Through Spontaneous  2.Through Process | |  | |  | |
| 515 | Did you take training disclosure? | 1.Yes 2. No | |  | |  | |

**Thank you**

**3.1.2. አማርኛ መጠይቅ**

**የስምምነት መጠየቂያ ቅጽ**

**በወላይታ ሶዶ ዩኒቨርስቲ የህክምናና ጤና ሳይንስ ኮሌጅ በማህበረሰብ አቀፍ ምርምር በወላይታ ዞን በኤች አይ ቪ ኤድስ ተጠቂ በሆኑ ህፃናት ላይ ራሳቸውን ስለማዎቅና ተዛማጅ ምክንያቶች ለማወቅ የተዘጋጀ መጠይቅ ነው፡፡**

**ውድ የጥናቱ ተሳታፊ በቅድሚያ እንደምን ዋሉ/አደሩ ስሜ ……... ይባላል እኔ በኤች አይ ቪ ኤድስ ክትትል ባሉ ህፃናት ላይ ለሚሰራ ጥናት መረጃ ሰብሳቢ ነኝ፡፡የጥናቱ ዓላማ በክትትል ላይ ያሉ ህፃናት ራሳቸውን ስለማዎቅና ተዛማጅ የሆኑ ምክንያቶችን በመለየት ላይ ያተኮረ ነው፡፡ ስለዚህ እርስዎ ለዚህ ጥናት ጠቃሚ የሆኑ መረጃዎችን ይሰጡናል ብለን እናስባለን ፡፡**

**በዚህ ሂደት እርስዎ የሚሰጡን ማንኛውም አይነት መረጃ በጥንቃቄና በምስጢር ስለሚያዝ እንዲሁም የእርስዎም ሆነ የልጅዎ ማንንነት አይመዘገብም ስለዚህ የሚሰጡን መረጃ ለልጅዎም ሆነ ለቤተሰቡ ለሚደረጉ ድጋፎች እንዲሁም የጤና ፖሊሲ ለሚነድፉ አካላት ግባት መሆኑን አውቀው ተአማኒነት ያለው እና ትክክለኛ መልስ እንዲሰጡን በታላቅ አክብሮት እንጠይቃለን ፈቃደኛ ከሆኑ**

**የአጥኝው ፊርማ …………………………**

**የተሳታፊው ፊርማ ………………….**

**የመረጃ ሰብሳቢው ፊርማ………….**

**መረጃው የተሰበሰበበት ቀን ………የተጀመረበት ሰዓት………የተጨረሰበት ሰዓት……….**

**ኮድ---------------------- የካርድ ቁጥር--------------**

**የመረጃ መሰብሰቢያ መጠይቅ**

**ክፍል 1፡- የልጁ(ቷ) ተንከባካቢ ማህበራዊና ስነ-ህዝብ ፀባይዎች መረጃ**

| **ተ.ቁ** | **ጥያቄ** | **ምላሽ** | **ኮድ** | **ዝለል** |
| --- | --- | --- | --- | --- |
| **101** | **ዕድሜ** | **-----------ዓመት** |  |  |
| **102** | **ፆታ** | **1 ወንድ 2. ሴት** |  |  |
| **103** | **ሃይማኖት** | **1.ኦርቶዶክስ 3. ፕሮቴስታንት**  **2.ሙስሊም 4. ካቶሊክ**  **5.ሌላ-----------** |  |  |
| **104** | **የጋብቻ ሁኔታ** | **1.ያገባ(ች) 2. ያላገባ(ች)**  **3. የፈታ(ች) 4. የሞተባት(በት)** |  |  |
| **105** | **የመኖሪያ ቦታ** | **1.ከተማ 2 ገጠር** |  |  |
| **106** | **የትምህርት ደረጃ** | **1.ማንበብና መፃፍ የማይችል**  **2.የመጀመሪያ ደረጃ (1-8)**  **3.ሁለተኛ ደረጃ (9-12)**  **4.ዲፕሎማ 5. ዲግሪ**  **6.ማስተርስ 7. ሌላ** |  |  |
| **107** | **የስራ ሁኔታ** | **1.ገበሬ 2. የመንግስት ሰራተኛ**  **3.ነጋዴ 4. የቀን ሰራተኛ**  **5.የቤት እመቤት 6. ሌላ-------** |  |  |
| **108** | **የቤተሰብ ብዛት** | **…………** |  |  |
| **109** | **ከልጁ(ቷ)ጋር ያለዎት ግንኙነት** | **1.አባት 2. እናት**  **3. ወንድም 4. እህት**  **5.ሌላ-------------** |  |  |
| **110** | **ወርኃዊ ገቢ** | **----------------------** |  |  |

**ክፍል ሁለት፡- የልጁ/ልጅቷ ማህበራዊና የስነ-ህዝብ ጸባይ መረጃ**

| **ተ.ቁ** | **ጥያቄ** | **መልስ** | **ኮድ** | **ዝለል** |
| --- | --- | --- | --- | --- |
| **201** | **ዕድሜ** | **……. ዓመት** |  |  |
| **202** | **ፆታ** | **1.ወንድ 2.ሴት** |  |  |
| **203** | **ሃይማኖት** | **1.ኦርቶዶክስ 2.ሙስሊም**  **3.ፕሮቴስታንት 4.ካቶሊክ**  **5.ሌላ** |  |  |
| **204** | **የክትትል ቦታ** | **1.ጤና ጣቢያ 2.ሆስፒታል** |  |  |
| **205** | **የትምህርት ደረጃ** | **1.ማንበብ እና መጻፍ ብቻ የሚችል**  **2.ኬጅ 3.የመጀመሪያ ደረጃ (1-8)**  **4. የሁለተኛ ደረጃ (9-12)**  **5. ሌላ** |  |  |
| **206** | **ከቤተሰቡ አባላት መካከል በኤች አይ ቪ ኤድስ**  **የሞተ ሰው አለ?** | **1.አዎ 2. የለም** |  | **መልሱ አይደለም ከሆነ**  **ወደ ቁ 301 ይሂዱ** |
| **207** | **ለጥያቄ ቁ. 206**  **መልሱ አዎ ከሆነ**  **ማንን?** | **1.እናት 2. አባት 3. ወንድም**  **4.እህት 5. ሁለቱንም (አባቱንም እናቱንም)** |  |  |

**ክፍል 3 ፡- የተንከባካቢው/ዋ ጤና ነክ ባህሪያት**

| **301** | **እርስዎስ ራስዎን ያውቃሉ (ተመርምረዋል?)** | **1.አዎ 2.የለም** |  | **መልሱ ሁለት ከሆነ ወደ ጥያቄ ቁ.**  **401 ይሂዱ** |
| --- | --- | --- | --- | --- |
| **302** | **መልስዎ አዎ ከሆነ የምርመራ ውጤቱ ምን ነበር** | **1. ፖዘቲቭ 2. ነጋቲቭ** |  |  |
| **303** | **መልሱ አንድ ከሆነ ለማን ተናግረዋል?** | **1.ለባለቤቴ 2. ለእናቴ**  **3.ለአባቴ 4. ለልጄ**  **5.ለጓደኛየ 6. ለወንድሜ**  **7.ለእህቴ 8. ለዘመዶቼ**  **9.ለሃይማኖት አባት**  **10. ማንም አያውቅም**  **11.ሌላ-------------** |  |  |
| **304** | **የኤችአይቪ ኤድስ መድኃኒት ጀምረዋል?** | **1.አዎ 2. አልጀመርኩም** |  |  |
| **305** | **የኤች አይ ቪ መድኃኒት ከጀመሩ ምን ያህል ጊዜ ሆነዎት?** | **---------------- ዓመት** |  |  |
| **306** | **ከቤተሰቡ መካከል ምን ያህሎቹ ከቫይረሱ ጋር ይኖራሉ?** | **--------------------** |  |  |
| **307** | **ካሉስ ማን ማን?** | **1.እናት 2. አባት**  **3.እህት 4. ወንድም**  **5.ሌላ** |  |  |

**ክፍል 4 ፡- የልጁ/ጇ ጤና ነክ ባህሪያት**

| **401** | **በኤች አይ ቪ ኤድስ የምርመራ ውጤት ወቅት የልጁ እድሜ ስንት ነበር?** | **---------------------** |  |  |
| --- | --- | --- | --- | --- |
| **402** | **የኤች አይ ቪ ኤድስ መድኃኒት ለምን ያህል ጊዜ ወስዷል/ለች?** | **………. ዓመት** |  |  |
| **403** | **ልጁ(ቷ) ከኤችአይቪ ኤድስ መድኃኒት በተጨማሪ ሌላ መድኃኒት ወስዷል?** | **1.አዎ 2. አልዎሰደም** |  | **መልሱ አይደለም ከሆነ ወደ ጥያቄ ቁጥር 405 ይሂዱ** |
| **404** | **የጥያቄ ቁ. 403 መልሱ አዎ ከሆነ ምን ዓይነት (የምን) መድኃኒት?** | **1. የቲቪ መድኃኒት**  **2. የልብ መድኃኒት**  **3. የግፊት መድኃኒት**  **4. የስኳር መድኃኒት**  **5. ሌላ-------------** |  |  |
| **405** | **ለልጁ(ቷ) መድኃኒት ኃላፊነት የሚወስደው ማን ነው?** | **1.እናት 2. አባት 3. ወንድም 4. እህት 5. ልጁ(ቷ)**  **6.ሌላ------------------** |  |  |
| **406** | **ልጁ(ቷ) ከዚህ በፊት ሆስፒታል ገብቶ(ተኝቶ) ያውቃል?** | **1.አዎ 2. የለም** |  | **መልሱ አይደለም ከሆነ ወደ ጥያቄ ቁጥር 408 ይሂዱ** |
| **407** | **ለጥያቄ ቁ.408 መልሱ አዎ ከሆነ ስንት ጊዜ** | **…………………………** |  |  |
| **408** | **ለልጁ(ቷ) ድጋፍ የሚያደርግ ድርጅት አለ?** | **1.አዎ 2. የለም** |  |  |
| **409** | **ለጥያቄ ቁ.410 መልሱ አዎ ከሆነ ከየት** | **1.የመንግስት ድርጅት**  **2.መንግስታዊ ያልሆነ ድርጅት**  **3.ሌላ--------------------** |  |  |
| **410** | **ምን አይነት ድጋፍ?** | **1.የምክር 2. የገንዘብ**  **3.የቁሳቁስ**  **4. ሌላ-----------------** |  |  |
| **411** | **ልጁ /ልጅቷ በዓለም ጤና ድርጅት የኤች አይ ቪ ኤድስ አከፋፈል መሰረት የጤና ደረጃው ስንት ነው?** | **1.ደረጃ-1 2. ደረጃ-2**  **3.ደረጃ-3 4. ደረጃ-4** |  |  |
| **412** | **ልጁ/(ቷ) በተጓዳኝ በሽታ ተጠቅቶ ያውቃል?** | **1.አዎ 2. የለም** |  |  |
| **413** | **አሁን ያለው የኤች አይ ቪ ቫይረስ ጭነት** | **---------------------** |  |  |
| **414** | **ህጸኑ አሁን የሚኖረው ከማን ጋር ነው?** | **1.ወላጅ 2. ወንድም**  **3.እህት 4. ዘመድ**  **5.ሌላ--------------------** |  |  |
| **415** | **መጀመሪያ የነበረው የሲዲ ፎር ቁጥር** | **-----------------------** |  |  |
| **416** | **መድኃኒት በትክክል ይወስዳል/አይወስድም/አትወስድም** | **1.በጥሩ ሁኔታ**  **2.በተመጣጣኝ**  **3.ብዙም አይወስድም** |  |  |
| **417** | **የሚወስደው የመድኃኒት ዓይነት/ሪጅመን/** | **---------------------** |  |  |
| **418** | **ህጻኑ በምግብ ተጎድቷል/አልተጎዳም** | **1.አዎ 2. የለም** |  |  |
| **419.** | **መልስዎ አዎ ከሆነ የጉዳቱ ዓይነት** | **1.ቀላል**  **2.ከባድ**  **3.በጣም ከባድ** |  |  |

**ክፍል 5 ፡- ኤችአይቪ ኤድስ ያለባቸው ህፃናት በኤች አይ ቪ ኤድስ እንዳለባቸው ከማወቅ ጋር የተያያዙ መረጃዎች**

| **501** | **ልጁ(ቷ) ኤች አይ ቪ ኤድስ እንዳለባት ታውቃለች? ያውቃል?** | **1.አዎ 2. የለም** |  | **መልሱ አይደለም ከሆነ ወደ ጥያቄ 502 ይሂዱ** |
| --- | --- | --- | --- | --- |
| **502** | **መልሱ አዎ ከሆነ በስንት አመቱ(ቷ)?** | **…………. ዓመት** |  |  |
| **503** | **ልጁ(ቷ) ኤች አይቪ ኤድስ እንዳለባት(እንዳለበት) ማን ነገረው(ራት)?** | **1.እናት 2. አባት**  **3. እህት 4. ወንድም**  **5.ዘመድ**  **6. የጤና ባለሙያዎች**  **7.ሌላ** |  |  |
| **504** | **ልጁ(ቷን) ኤች አይ ቪ ኤድስ እንዳለባት(እንዳለበት) ለመንገር ለምን ወሰኑ?** | **1.ልጁ (ቷ) ማዎቅ ያለባት እድሜ ላይ ስለሆነች**  **2.በልጁ(ቷ)የተደጋገመ ጥያቄ**  **3.ልጁ(ቷ)ህክምናውን (መድሃኒቱን) እንድትዎስድ(ድ)ለማድረግ**  **4.ራሱን እንዲጠብቅ እና ሳታውቅ(ሳያውቅ)ለሌሎች ማስተላለፍን ለመከላከል**  **5.በድንገት እንዳታውቅ(እንዳያውቅ) ብየ ስለምፈራ**  **6.ሌላ------------------** |  |  |
| **505** | **ካላዎቀች(ካላዎቀ) ለምን አላሳዎቁም ምክንያትዎ ምንድን ነው?** | **1.ልጁ(ቷ)በጣም ትንሽ ስለሆነች**  **2.ልጁ(ቷ) መድኃኒቱን አልዎስድም እንዳትል (እንዳይል) ብየ ስለምፈራ**  **3.በልጁ(ቷ) ወይም ቤተሰቡ ማህበራዊ ተቀባይነት ያጣል ብየ ስለምፈራ**  **4.ልጁ(ቷ)ምስጢር መጠበቅ አትችልም(አይችልም)ብየ ስለምፈራ**  **5.ልጁ(ቷ) ኤችአይቪ ኤድስ**  **እንዳለባት(እንዳለበት) የማያውቅ የቤተሰብ አባል ስላለ ችግር ይፈጠራል**  **6.ከቤተሰቡ ጋር ያለው ጥሩ ግንኙነት ይቋረጣል ብየ ስለምፈራ**  **7.የልጁ(ቷ)ስሜት ስነ ልቦና ይጎዳል ብየ ስለምፈራ**  **8.ሌላ-----------------------** |  |  |
| **506** | **ልጁ(ቷ) ኤችአይቪ ኤድስ**  **እንዳለባት(እንዳለበት) ካላዎቀች (ቀ) ወደ ጤና ተቋም ምን ብለው ይወስዱታል/ይወስዷታል?** | **1.የቲቪ ህክምና**  **2.ለሳምባ ምች ህክምና**  **3.ለቀጠሮ**  **4.መድኃኒት ልናመጣ**  **5.ሌላ -------------** |  |  |
| **507** | **ልጁ(ቷ)ኤች አይ ቪ ኤድስ እንዳለባት (እንዳለበት) የማሳወቅ ኃላፊነትአለብኝ ብለዉ ያስባሉ?** | **1.አዎ 2. የለም** |  |  |
| **508** | **ለዎደፊት ለልጅዎ ኤችአይቪ እንዳለባት (እንዳለበት**  **ለማሳዎቅ እቅድ አለዎት** | **1.አዎ 2. የለኝም** |  |  |
| **509** | **ለጥያቄ ቁጥር 508 መልሱ አዎ ከሆነ በስንት ዓመቱ?** | **……………….** |  |  |
| **510** | **ለልጁ(ቷ)ኤችአይቪ እንዳለባት (እንዳለበት የማሳወቅ ኃላፊነት ለማነው ብለው ያስባሉ?** | **1.እናት 2. አባት 3. አያት**  **4.የጤና ባለሙያዎች**  **5.ሌላ------------------** |  |  |
| **511** | **በሽታውን ለሕጻኑ በሚገልጹበት ጊዜ ያለወት የመተማመን ስሜት** | **1.እርግጠኛ**  **2.በከፊል**  **3.እርግጠኛ መተማመን አልነበረኝም** |  |  |
| **512** | **በሽታውን በሚገልጹበት ጊዜ ከጤና ባለሙያው ድጋፍ አግኝተዋል** | **1.አዎ**  **2.የለም** |  |  |
| **513** | **በሽታውን ለሕጻኑ በምን መልኩ ገለጹለት** | **1.በሙሉ 2.በከፊል**  **3.በሌላ በማስመሰል** |  |  |
| **514** | **ልጁ/ቷ እንዴት አወቀ/ች** | **1.በድንገት 2.በሂደት** |  |  |
| **515** | **ልጅዎት ከቫይረሱ ጋር መኖሩን ለማሳዎቅ ስልጠና ተሰጥቷችሁ ያውቃል** | **1.አዎ 2.የለም** |  |  |

**እናመሰግናለን**
