# Supplementary material for: Factors associated with children’s HIV- positive status disclosure in Wolaita Zone, Southern Ethiopia: a cross-sectional study
Source: Ital J Pediatr. 2022 Jun 6;48:86. doi: 10.1186/s13052-022-01287-6 (PMC9169329; doi:10.1186/s13052-022-01287-6)
Supplement: Supplementary file 2 — Additional file 2: Table S1. Socio-demographic and clinical characteristics of caregivers in Wolaita Zone, Southern Ethiopia, 2021 (n=203). [file 13052_2022_1287_MOESM2_ESM.docx]

Table 1:- Socio-demographic and clinical characteristics of caregivers in Wolaita Zone, Southern Ethiopia, 2021 (n=203).

| Variables | Category | Frequency (n) | Percent (%) |
| --- | --- | --- | --- |
| Age | <30 Year | 29 | 14.3 |
|  | 30-44 Year | 113 | 55.7 |
|  | ≥45 Year | 61 | 30.0 |
| Sex | Female | 172 | 84.7 |
|  | Male | 31 | 15.3 |
| Marital Status | Married | 98 | 48.3 |
|  | Unmarried | 105 | 51.7 |
| Educational status | No formal education | 78 | 38.4 |
|  | Elementary (1-8) | 57 | 28.1 |
|  | High school (9-12) | 50 | 24.6 |
|  | College & above | 18 | 8.9 |
| Residency | Urban | 188 | 92.6 |
|  | Rural | 15 | 7.4 |
|  |  |  |  |
|  |  |  |  |
| Relationship with children | First degree relatives | 176 | 86.7 |
|  | Second degree relatives | 27 | 13.3 |
| HIV status | Positive | 144 | 70.9 |
|  | Negative | 59 | 29.1 |
| Duration on ART | < 24 months | 124 | 61.1 |
|  | ≥ 24 months | 79 | 38.9 |
