# Supplementary material for: Factors associated with children’s HIV- positive status disclosure in Wolaita Zone, Southern Ethiopia: a cross-sectional study
Source: Ital J Pediatr. 2022 Jun 6;48:86. doi: 10.1186/s13052-022-01287-6 (PMC9169329; doi:10.1186/s13052-022-01287-6)
Supplement: Supplementary file 3 — Additional file 3: Table S2. Socio-demographic and clinical characteristics of children in Wolaita Zone, Southern Ethiopia, 2021 (n=203). [file 13052_2022_1287_MOESM3_ESM.docx]

Table 2:- Socio-demographic and clinical characteristics of children in Wolaita Zone, Southern Ethiopia, 2021 (n=203).

| Variables | Category | Frequency (n) | Percent (%) |
| --- | --- | --- | --- |
| Age | <12 | 112 | 55.2 |
|  | ≥12 | 91 | 44.8 |
| Sex | Male | 93 | 45.8 |
|  | Female | 110 | 54.2 |
| Educational status | Not started education | 23 | 11.3 |
|  | Start education | 180 | 88.7 |
| Death of any of his/her parent | Yes | 70 | 34.5 |
|  | No | 133 | 65.5 |
| Age at diagnosis of HIV | <4 year | 109 | 53.7 |
|  | ≥4 year | 94 | 46.3 |
| Duration on ART | <72 months | 116 | 57.1 |
|  | ≥72 months | 87 | 42.9 |
| History of ART interruption and restart | No  Yes | 168  35 | 82.8  17.2 |
| WHO clinical stage | Stage I/II | 192 | 94.6 |
|  | Stage III/IV | 11 | 5.4 |
| History of opportunistic infection | Yes | 70 | 34.5 |
|  | No | 133 | 65.5 |
| History of Hospitalization | Yes | 77 | 37.9 |
|  | No | 126 | 62.1 |
| ART adherence level | Good | 181 | 89.2 |
|  | Fair/poor | 22 | 10.8 |
| With whom currently living | Biological parents | 178 | 87.7 |
|  | Other relatives | 25 | 12.3 |
| Types of health facility | Health Center | 78 | 38.4 |
|  | Hospital | 125 | 61.6 |

Abbreviations: WHO, World Health Organization; ART, Antiretroviral Therapy
