# Supplementary material for: Factors associated with children’s HIV- positive status disclosure in Wolaita Zone, Southern Ethiopia: a cross-sectional study
Source: Ital J Pediatr. 2022 Jun 6;48:86. doi: 10.1186/s13052-022-01287-6 (PMC9169329; doi:10.1186/s13052-022-01287-6)
Supplement: Supplementary file 4 — Additional file 4: Table S3. Bivariable and multivariable logistic regression analysis of factors associated with HIV positive status disclosure among HIV–infected children in Wolaita Zone, Sothern Ethiopia, 2021 (n=203). [file 13052_2022_1287_MOESM4_ESM.docx]

Table 3:- Bivariable and multivariable logistic regression analysis of factors associated with HIV positive status disclosure among HIV–infected children in Wolaita Zone, Sothern Ethiopia, 2021 (n=203).

| Factors | Disclosure status n (%) | |  |  |  |  |
| --- | --- | --- | --- | --- | --- | --- |
|  | Not disclosed | Disclosed | COR (95% CI) | AOR (95% CI) | P-value |  |
| Age of child |  |  |  |  |  |  |
| <12 year | 34 (30.4) | 78 (69.6) | 1 | 1 |  |  |
| ≥12 year | 7 (7.7) | 84 (92.3) | 6.21 (3.03-9.73) | 7.54 (2.87-15.62) ** | <0.001 |  |
| Child’s educational Status |  |  |  |  |  |  |
| Not started education | 11 (47.8) | 12 (52.2) | 1 | 1 |  |  |
| Start education | 55 (30.6) | 125 (69.4) | 1.79 (0.89-3. 87) | 1.75 (0.45 -2.83 ) | 0.61 |  |
| Residency |  |  |  |  |  |  |
| Urban | 60 (31.9) | 128 (68.1) | 1.28 (0.61 -3.95) | 1.19 (0.47 -3.62) | 0.42 |  |
| Rural | 3 (20.0) | 12 (80.0) | 1 | 1 |  |  |
| Death of any of his/her parent |  |  |  |  |  |  |
| Yes  No | 18 (25.7)  107 | 52 (74.3)  171 | 1.48 (1. 17-2.85 7)  1 | 1.96 (1.05-3.84 ) **  1 | 0.02 |  |
| HIV status care givers |  |  |  |  |  |  |
| Positive | 54 (37.5) | 90 (62.5) | 2.47 (1.56-4.77) | 2.44 (0.63-2.05) | 0.16 |  |
| Negative | 14 (23.7) | 45 (76.3) | 1 | 1 |  |  |
| Unknown |  |  |  |  |  |  |
| Child’s duration on ART |  |  |  |  |  |  |
| < 72 months | 54 (46.6) | 62 (53.4) | 1 | 1 |  |  |
| ≥ 72 months | 17 (19.5) | 70 (80.5) | 3.43 (2.59-5.79) | 3.84 (1.65-6.72) ** | 0.01 |  |
| Age of child at HIV diagnosis |  |  |  |  |  |  |
| < 4 year | 47 (43.1) | 62 (56.9) | 3. 18 (1.79-4.37) | 1. 26 (0.86-3.72) | 0.85 |  |
| ≥ 4 year | 24 (25.5) | 70 (74.5) | 1 | 1 |  |  |
| Current care giver |  |  |  |  |  |  |
| Biological parent | 65 (36.5) | 113 (63.5) | 1 | 1 |  |  |
| Other relatives | 4 (13.0) | 21 (84.0) | 3.9 (1.45-9.63) | 4.59 (0.93-11.76) | 0.52 |  |
| Relationship with children |  |  |  |  |  |  |
| First degree relative | 64 (36.4) | 112 (63.6) | 0.47 (0.35 -1.13 ) | 2.08 (0.76-7. 91) | 0.29 |  |
| Second degree relative | 7 (25.9) | 20 (74.1) | 1 | 1 |  |  |
| Types of health facility |  |  |  |  |  |  |
| Health Center | 61(78.2) | 17(21.8) | 1 | 1 |  |  |
| Hospital | 59(47.2) | 66(52.8) | 0.48(0.27-0.81) | 2.13 (1.33-4.67)** | <0.001 |  |

**Notes:** **p-value < 0.05(statistically significant association), 1: reference category.

**Abbreviations:** AOR: Adjusted odd ratio; CI: Confidence Interval; COR: Crud odd ratio
